# Biological, pharmacological and immunological activities of novel 6-amino-acid-substituted 14-alkoxy-*N*-methylmorphinans

**DOI:** 10.1186/1471-2210-11-S2-A5

**Published:** 2011-09-05

**Authors:** Elena Guerrieri, Valeria Follia, Dorota Garczarczyk, Silvia B Rief, Monika Fink, Muhammad F Asim, Mariana Spetea, Helmut Schmidhammer

**Affiliations:** 1 Department of Pharmaceutical Chemistry, Institute of Pharmacy and Center for Molecular Biosciences, University of Innsbruck, 6020 Innsbruck, Austria

## Background

Of the three opioid receptors, μ (MOP), δ (DOP) and κ (KOP), the MOP type is the most involved in the action of opioids in the gut. Experimental studies on models of intestinal inflammation and inflammatory bowel disease (IBD) support an anti-inflammatory role of peripheral MOP receptors in the gut, besides their involvement in pain control (analgesia) and gastrointestinal motility (anti-diarrheal effects). Research focuses increasingly on exploring the therapeutic potential of peripheral MOP receptors aiming for identification of peripheral ligands as improved treatment for debilitating conditions associated with bowel functions. One strategy to increase peripheral selectivity includes chemical modifications that enhance hydrophilicity [1,2]. Our work in the field of peripherally acting opioids has led to a series of opioids with zwitterionic moieties (i.e. amino acid residues) attached to the C-6 position of 14-*O*-methyloxymorphone, which may represent novel therapeutic molecules for IBD. These 14-alkoxymorphinans were pharmacologically and immunologically characterized.

## Methods

Synthesis of novel zwitterionic 14-alkoxymorphinans was accomplished by multi-step syntheses. Binding, functional and immunomodulatory activities were determined *in vitro*. Antinociceptive activities were assessed using acetic acid-induced writhing and tail-flick tests. Physicochemical properties (logP and logD) were determined using the MarvinSketch software.

## Results

*In vitro*, the new 6-amino-acid-substituted 14-alkoxymorphinans bound with high affinity and showed agonist activity towards the MOP receptor. They significantly inhibited the nuclear transcription factor kappaB (NF-κB) activation in tumor-necrosis factor-α (TNF-α) and lipopolysaccharide (LPS)-stimulated human monocytic THP-1 cells. *In vivo*, they produced dose-dependent antinociceptive effects in mice after subcutaneous administration, being several-fold more potent than morphine. Based on the calculated logP and logD values, an increase of hydrophilicity, and thus peripheral selectivity can be achieved by attachment of amino acid residues to the morphinan skeleton.

## Conclusions

Novel MOP agonists acting in the periphery with combined immunosuppressive and analgesic properties may provide a new approach for the treatment of IBD.
